# Utilization of different MurNAc sources by the oral pathogen *Tannerella forsythia* and role of the inner membrane transporter AmpG

**DOI:** 10.1186/s12866-020-02006-z

**Published:** 2020-11-17

**Authors:** Valentina M. T. Mayer, Markus B. Tomek, Rudolf Figl, Marina Borisova, Isabel Hottmann, Markus Blaukopf, Friedrich Altmann, Christoph Mayer, Christina Schäffer

**Affiliations:** 1 Department of NanoBiotechnology, NanoGlycobiology unit, Universität für Bodenkultur Wien, Vienna, Austria; 2 Department of Chemistry, Institute of Biochemistry, Universität für Bodenkultur Wien, Vienna, Austria; 3 Department of Biology, Eberhard Karls Universität Tübingen, Microbiology/Glycobiology, Interfaculty Institute of Microbiology and Infection Medicine Tübingen, Tübingen, Germany; 4 Department of Chemistry, Institute of Organic Chemistry, Universität für Bodenkultur Wien, Vienna, Austria

**Keywords:** *Tannerella forsythia*, Peptidoglycan, Muropeptides, AmpG permease, *N*-acetylmuramic acid sources, Anhydro-*N*-acetylmuramic acid, Oral biofilm

## Abstract

**Background:**

The Gram-negative oral pathogen *Tannerella forsythia* strictly depends on the external supply of the essential bacterial cell wall sugar *N*-acetylmuramic acid (MurNAc) for survival because of the lack of the common MurNAc biosynthesis enzymes MurA/MurB. The bacterium thrives in a polymicrobial biofilm consortium and, thus, it is plausible that it procures MurNAc from MurNAc-containing peptidoglycan (PGN) fragments (muropeptides) released from cohabiting bacteria during natural PGN turnover or cell death. There is indirect evidence that in *T. forsythia*, an AmpG-like permease (*Tanf_08365*) is involved in cytoplasmic muropeptide uptake. In *E. coli*, AmpG is specific for the import of *N*-acetylglucosamine (GlcNAc)-anhydroMurNAc(−peptides) which are common PGN turnover products, with the disaccharide portion as a minimal requirement. Currently, it is unclear which natural, complex MurNAc sources *T. forsythia* can utilize and which role AmpG plays therein.

**Results:**

We performed a screen of various putative MurNAc sources for *T. forsythia* mimicking the situation in the natural habitat and compared bacterial growth and cell morphology of the wild-type and a mutant lacking AmpG (*T. forsythia* Δ*ampG*). We showed that supernatants of the oral biofilm bacteria *Porphyromonas gingivalis* and *Fusobacterium nucleatum*, and of *E. coli* Δ*ampG*, as well as isolated PGN and defined PGN fragments obtained after enzymatic digestion, namely GlcNAc-anhydroMurNAc(−peptides) and GlcNAc-MurNAc(−peptides), could sustain growth of *T. forsythia* wild-type, while *T. forsythia* Δ*ampG* suffered from growth inhibition. In supernatants of *T. forsythia* Δ*ampG*, the presence of GlcNAc-anhMurNAc and, unexpectedly, also GlcNAc-MurNAc was revealed by tandem mass spectrometry analysis, indicating that both disaccharides are substrates of AmpG. The importance of AmpG in the utilization of PGN fragments as MurNAc source was substantiated by a significant *ampG* upregulation in *T. forsythia* cells cultivated with PGN, as determined by quantitative real-time PCR. Further, our results indicate that PGN-degrading amidase, lytic transglycosylase and muramidase activities in a *T. forsythia* cell extract are involved in PGN scavenging.

**Conclusion:**

*T. forsythia* metabolizes intact PGN as well as muropeptides released from various bacteria and the bacterium*’*s inner membrane transporter AmpG is essential for growth on these MurNAc sources, and, contrary to the situation in *E. coli*, imports both, GlcNAc-anhMurNAc and GlcNAc-MurNAc fragments.

## Background

*Tannerella forsythia* is a Gram-negative, obligate anaerobic bacterium inhabiting the oral cavity that is strongly associated with periodontitis (gum disease) [1], a multifactorial, inflammatory biofilm disease affecting the tissues supporting the teeth, ultimately leading to tooth loss [2]. The disease is widespread among the adult population worldwide and its cost estimate was up to 396 billion USD in 2015 [3]. There is also mounting evidence of a link between periodontitis and various systemic medical conditions, including, e.g., cardiovascular diseases, rheumatoid arthritis, and Alzheimer’s disease [4, 5]. *T. forsythia* is usually found alongside the Gram-negatives *Porphyromonas gingivalis* and *Treponema denticola*, together constituting the “red complex” of periodontal pathogens and acting as late colonizers of a dysbiotic polymicrobial biofilm, which causes periodontitis [6, 7]. Other crucial colonizers of this oral biofilm (commonly known as dental plaque) are streptococci adhering to mucosal surfaces and the “bridge organism” *Fusobacterium nucleatum* [8].

Among the dental plaque bacteria, *T. forsythia* has a unique status, because of its auxotrophy for the commonly essential bacterial peptidoglycan (PGN) cell wall sugar *N*-acetylmuramic acid (MurNAc) and, thus, strictly depends on external supply of MurNAc for growth and maintenance of cell morphology, when grown in monospecies laboratory culture [9]. We recently confirmed by scanning electron microscopy the transition from healthy, rod-shaped to fusiform *T. forsythia* cells upon stepwise MurNAc depletion [10]. It is important to consider that growth in monospecies culture does not reflect the situation in the oral cavity, where *T. forsythia* is part of the polymicrobial biofilm consortium. Recently it was found that a pool of muropeptides from *F. nucleatum* could replace free MurNAc to promote growth of *T. forsythia* [11].

*T. forsythia’s* MurNAc auxotrophy is due to the absence of the genes for the PGN biosynthesis enzymes MurA and MurB in its genome [12]; these are required for the de novo synthesis of uridyl-diphosphate (UDP) activated MurNAc for subsequent channelling into the PGN biosynthesis route [13]. PGN is a bag-shaped macromolecule (PGN sacculus) external to the bacterial cytoplasmic membrane that serves as a protection from adverse environmental effects and disruption due to high internal osmotic pressure and, thus, is essential for bacterial survival [14]. Despite its predictably unique PGN metabolism, *T. forsythia* possesses a common Gram-negative PGN structure of the A1γ-type [15] as was recently published by our group [16]: MurNAc and β-1,4-linked GlcNAc alternatingly form glycan backbone strands, terminating with a non-reducing 1,6-anhydroMurNAc residue (anhMurNAc) at the reducing end, with stem peptides composed of alanine (Ala), glutamic acid (Glu), *meso*-diaminopimelic acid (*m-*DAP), and Ala, and likely a direct cross-linkage between *m-*DAP on the third and Ala on the fourth position of cross-linked stem peptides. This PGN type occurs also in *P. gingivalis* [16] and *Escherichia coli* [13], while in the PGN of *F. nucleatum*, *m*-DAP is replaced by lanthionine [17].

In the course of the natural cell wall turnover, bacteria enzymatically digest their PGN [18]. The PGN backbone may be cleaved by *N*-acetylmuramidases cutting the β-1,4 glycosidic bond between MurNAc and GlcNAc, and lytic transglycosylases, which concomitantly introduce a 1,6-anhydro bond at the MurNAc residue [13, 19]. Endo-acting *N*-acetylglucosaminidases catalyse the hydrolysis of the other glycosidic bond (GlcNAc-β-1,4-MurNAc), thus, generating distinct MurNAc-GlcNAc-peptides [20]. Accrued PGN fragments (muropeptides) are either recycled or released into the environment, where they are thought to get accessible for cohabiting bacteria [11].

For PGN turnover and recycling [21], many Gram-negatives involve lytic transglycosylases generating GlcNAc-anhMurNAc-peptides (anhydromuropeptides) and (AmpG-like) permeases to transport these anhydromuropeptides across the cytoplasmic membrane. AmpG was originally identified in *E. coli* as a regulator necessary for the induction of the β-lactamase AmpC [18]. Its function as a PGN-recycling permease with likely specificity for GlcNAc-anhMurNAc(−peptides) was shown by Cheng and Park [22]. The authors detected inhibition of the uptake of radiolabelled GlcNAc-anhMurNAc and GlcNAc-anhMurNAc-peptides by an *E. coli* Δ*ampG* deletion mutant in comparison to the wild-type. By adding carbonyl cyanide 3-chlorophenylhydrazone, the uptake in the wild-type was decreased to a similar level as in the mutant, showing the transporter’s dependence on a proton motive force. Regarding other substrates, including GlcNAc-MurNAc(−peptides) and anhMurNAc(−peptides), no significant difference was found between wild-type and the Δ*ampG* mutant. The genome of *T. forsythia* encodes one muropeptide permease AmpG homologue (*Tanf_08365*), which displays 27% amino acid identity with the *E. coli* permease and is located in the middle of a nine-genes spanning genomic cluster (*Tanf_08345* to *Tanf_08385*) representing a single transcriptional unit  [11]. This gene cluster comprises several putative PGN recycling genes, including the already characterized genes for the MurNAc transporter MurT [23], the kinase MurK, and the etherase MurQ [10], which work in concert along the anabolic UDP-MurNAc recycling pathway [21]. The function of *Tanf_08365* as a muropeptide transporter was recently indirectly confirmed by heterologous expression in *E. coli* applying a reporter system with a muropeptide inducible β-lactamase reporter gene [17]. A plasmid containing the inducible reporter gene was introduced to an *E. coli* double mutant lacking the muropeptide transporter AmpG and the cytosolic amidase AmpD. The complementation of this *E. coli* reporter strain with the *T. forsythia ampG* gene restored the uptake of PGN recycling products, and due to the resulting cytosolic accumulation of anhMurNAc-peptides, induction of β-lactamase activity could be measured. Furthermore, a direct regulation of the implied operon by the hybrid two component system GppX was revealed, since the expression of GppX (*Tanf_13760*) led to a seven-fold higher induction of the promotor region of the operon and *Tanf_08365* was significantly downregulated in a *T. forsythia gppX* deletion mutant [11, 24]. Notably, growth of the *gppX* mutant was not completely abolished on either muropeptides or free MurNAc, suggesting that the *ampG-*containing operon is expressed constitutively at basal levels [11].

In this present study, various MurNAc sources of different complexity likely present in the polymicrobial biofilm setting in the oral habitat were analysed for their suitability to sustain growth of the periodontal pathogen *T. forsythia*. Further, the role and specificity of the AmpG permease in the utilization of these putative MurNAc substitutes was assessed. Specifically, this included (i) preparation of MurNAc sources from oral bacteria and from *E. coli*, followed by compositional analysis by HPAEC-PAD, electrospray ionization LC-ESI-MS, and MS^2^, (ii) chemical synthesis of anhMurNAc, (iii) monitoring of growth and cell morphology using SEM, as well as (iv) comparing the effects of the different MurNAc sources in *T. forsythia* wild-type versus a novel isogenic *T. forsythia* Δ*ampG* mutant to unravel the role and specificity of the cytoplasmic membrane transporter AmpG in the PGN metabolism. Overall, this study sheds light on the metabolic strategies the periodontal pathogen *T. forsythia* has elaborated to build up its cell wall PGN.

## Results

### Preparation and analysis of putative MurNAc sources

To obtain insight into which complex MurNAc sources likely present in the oral habitat *T. forsythia* can metabolize to compensate for its MurNAc auxotrophy, different MurNAc sources mimicking the circumstances in the oral biofilm were prepared and their composition was analysed.

Considering *T. forsythia* as part of the oral biofilm consortium, supernatants from the red complex member *P. gingivalis* W83 and the “bridge organism” *F. nucleatum* OMZ 589, as well as *E. coli* MC4100 and *E. coli* TP72 (Δ*ampG*), were harvested at OD_600_ ~ 2.0. Supernatants were analysed by LC-ESI-MS, using an amaZon ion trap mass spectrometer, and data were verified by MS^2^ analysis, using standards for MurNAc and anhMurNAc.

While no PGN fragments were detected in supernatants of *E. coli* MC4100 indicative of efficient PGN recycling, the supernatant of the *E. coli* Δ*ampG* mutant, which is unable to take up its PGN recycling products due to deficiency in *ampG*, was confirmed to contain GlcNAc-anhMurNAc, as published previously (Supplemental Fig. S1A) [25]. In supernatants of *F. nucleatum* OMZ 589, MS analysis revealed the presence of GlcNAc-anhMurNAc indicative of inefficient PGN recycling. Conforming with the theoretical mass of *m/z* 479.19*,* the disaccharide was observed with a mass of *m/z* 479.29 [M + H]^+^ and verified by MS^2^ analysis (Supplemental Fig. S1B). The analysis of the *P. gingivalis* supernatant revealed to be more difficult. However, after enrichment via hydrophobic interaction liquid chromatography-solid phase extraction (HILIC-SPE), the presence of GlcNAc-MurNAc could be detected. Conforming with the theoretical mass of *m/z* 497.19*,* the disaccharide was observed with a mass of *m/z* 497.18 [M + H]^+^ and verified by MS^2^ analysis (Supplemental Fig. S1C).

PGN sacculi as a putative source of MurNAc were isolated from *T. forsythia* wild-type, *P. gingivalis* W83*, F. nucleatum* OMZ 589, and *E. coli* MC4100, following a published protocol [26]. After digestion of PGN with the *N*-acetylmuramidase mutanolysin, the PGN composition was determined by LC-MS, revealing the presence of GlcNAc-MurNAc-peptides (G-M-tri/tetra) and dimers thereof as typical digestion products. For *E. coli* [13], as well as *T. forsythia* and *P. gingivalis*, the stem peptide is composed of Ala, Glu, *m-*DAP and Ala, referring to the PGN-type A1γ, as published previously [16]. Regarding the PGN of *F. nucleatum*, Vasstrand et al. reported that lanthionine instead of *m*-DAP occurs in the stem peptide [27, 28]. This result was confirmed by our study, providing the first MS data for *F. nucleatum* PGN. The observed peaks were *m/z* 889.32 [M + H]^+^ for G-M-tri and *m/z* 960.36 [M + H]^+^ for G-M-tetra, and, thus, in accordance with theoretical masses of *m/z* 889.33 and *m/z* 960.37 for lanthionine containing fragments (Supplemental Fig. S2).

The preparation of GlcNAc-MurNAc-peptides and GlcNAc-anhMurNAc-peptides was accomplished by digesting *E. coli* MC4100 PGN with mutanolysin and the lytic transglycosylase Slt70, respectively. Additional treatment of the digestion products with the amidase AmiD produced free disaccharides as confirmed by LC-MS (Supplemental Fig. S3), in addition to peptides.

Finally, anhMurNAc was chemically synthesized [29] and purified by HILIC, with an achieved overall yield of 5.4%. All analytical NMR data of anhMurNAc were in agreement with previously published data (Supplemental Fig. S4).

### Identification of MurNAc sources for *T. forsythia*

**Fig. 1:**
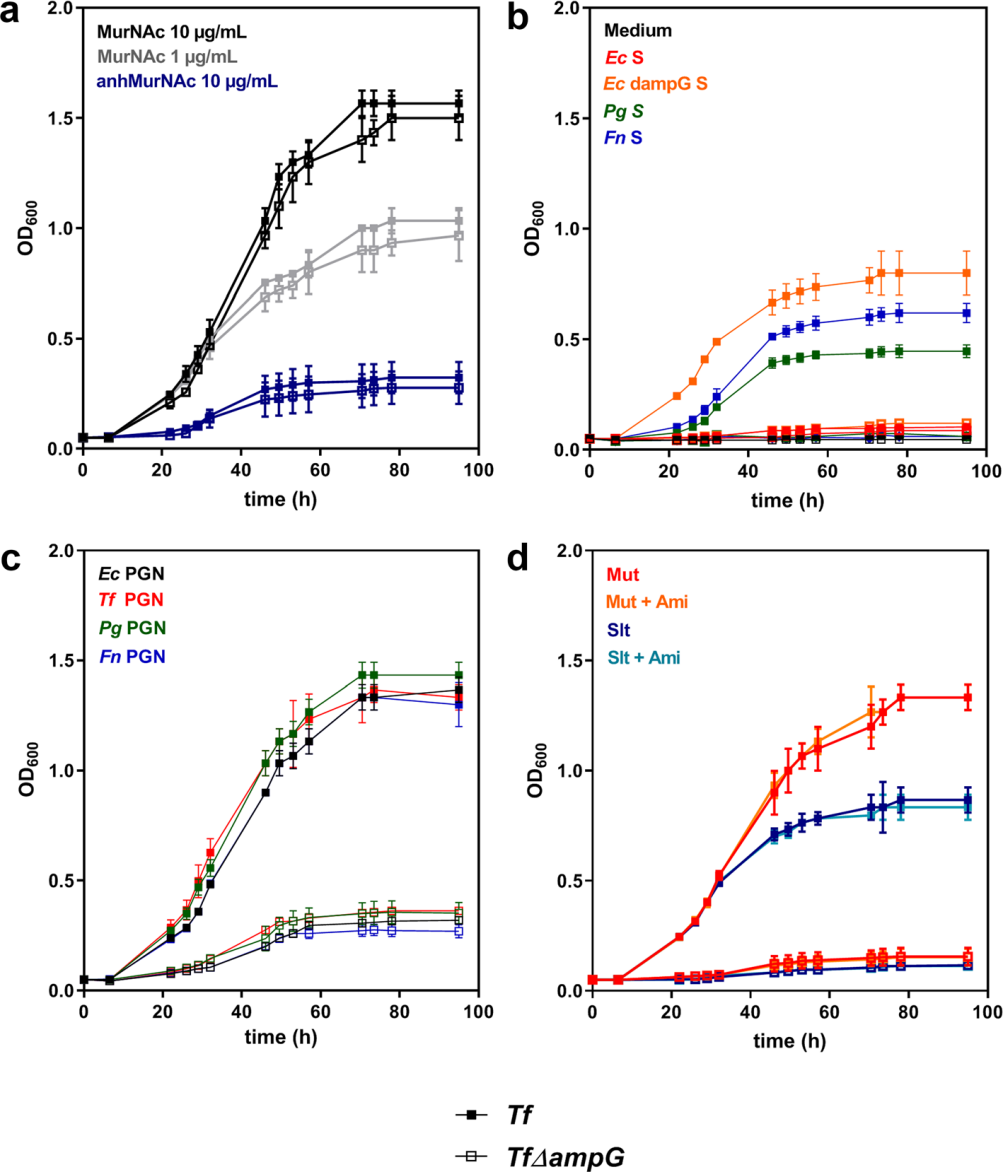
Growth of *T. forsythia* wild-type (*Tf;* solid symbols) and *T. forsythia* Δ*ampG* mutant (*Tf* Δ*ampG;* open symbols) with MurNAc and anhMurNAc (**a**) and different MurNAc substituents (color-coded), including supernatants of *E. coli* MC4100, *E. coli* Δ*ampG*, *P. gingivalis* W83 and *F. nucleatum* OMZ 589 (**b**); PGN of *E. coli*, *T. forsythia*, *P. gingivalis* and *F. nucleatum* (**c**); and digests of *E. coli* PGN with mutanolysin (Mut), yielding GlcNAc-MurNAc-peptides, and Slt70 (Slt), yielding GlcNAc-anhMurNAc-peptides, plus AmiD (+Ami), cutting off the peptides, yielding GlcNAc-MurNAc (**d**). The mean OD_600_ values of biological triplicates and standard errors are shown

The different putative MurNAc sources of known composition as determined above were added as a BHI medium supplement for *T. forsythia* wild-type and *T. forsythia* Δ*ampG*, instead of free MurNAc. Of note, *T. forsythia* is routinely grown with supplementation of 10 μg ml^− 1^ MurNAc, reaching a maximal OD_600_ of ~ 1.6, while supplementation of 1 μg ml^− 1^ MurNAc supports growth up to an OD_600_ of ~ 1.0 only (Fig. 1a). To correct for differences in the MurNAc concentration of the different sources, supernatants as well as PGN sacculi were hydrolysed and the MurNAc concentration was determined by HPAEC-PED. Since standards for MurNAc and anhMurNAc had the same retention time of 42.4 min, a combined value for both components was obtained (data not shown).

First, MurNAc was substituted by bacterial culture supernatants. Due to the low detectable amount of MurNAc/anhMurNAc in bacterial supernatants of *F. nucleatum*, *P. gingivalis* and *E. coli*, these were added in the most concentrated form possible (before reaching viscosity), equalling a MurNAc/anhMurNAc concentration in the growth medium below 1 μg ml^− 1^. While the supply of the GlcNAc-anhMurNAc-containing supernatant of *E. coli* Δ*ampG* yielded a *T. forsythia* culture of an OD_600_ of ~ 0.8, supernatants of *E. coli* wild-type could not sustain growth of *T. forsythia* wild-type due to the absence of any detectable muropeptides. Supernatants of *F. nucleatum*, also shown to contain GlcNAc-anhMurNAc, albeit in lower concentration (Supplemental Fig. S1), sustained growth of *T. forsythia* wild-type to an OD_600_ of ~ 0.6 and supernatants of *P. gingivalis,* containing minimal amounts of GlcNAc-MurNAc, sustained growth to an OD_600_ of ~ 0.45 (Fig. 1b).

**Fig. 2:**
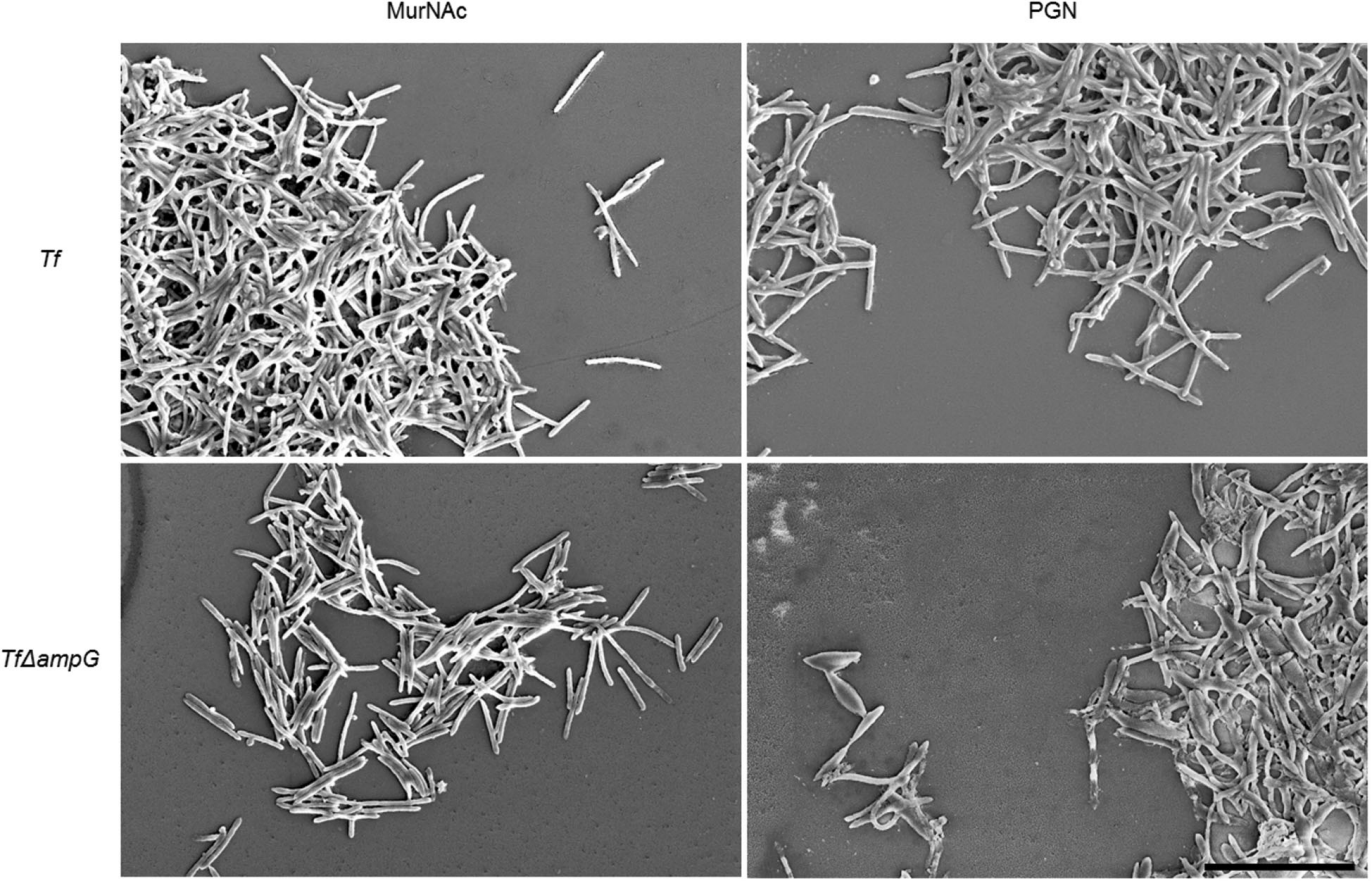
SEM micrographs of *T. forsythia* wild-type (*Tf*) and mutant *T. forsythia* Δ*ampG* (*Tf* Δ*ampG*), cultivated with MurNAc (first column) and with PGN (second column), showing the change of *T. forsythia* Δ*ampG* cell morphology from rod-shaped to fusiform cells when cultivated with PGN instead of MurNAc. Scale bar, 10 μm

Growth of *T. forsythia* wild-type with isolated PGN sacculi corresponding to a final MurNAc/anhMurNAc concentration in the growth medium of 1 μg ml^−1^was unrestrictedly sustained, independent of the bacterial source from which it was isolated from. Cultures yielded OD_600_ values of ~ 1.4 (Fig. 1c) and bacterial cells showed no alteration of cell morphology, according to SEM evidence (Fig. 2). The supply of specific digests of PGN by the *N*-acetylmuramidase mutanolysin, containing mainly GlcNAc-MurNAc-peptides, had a similar effect on growth of *T. forsythia*, reaching OD_600_ values of ~ 1.3. In comparison, digests of PGN by the lytic transglycosylase Slt70, containing mainly GlcNAc-anhMurNAc-peptides, restricted growth of *T. forsythia* wild-type to an OD_600_ of ~ 0.8. Additional treatment of PGN digests with amidase AmiD, producing free disaccharides and peptides, resulted in a comparable growth behaviour according to OD_600_ measurement of *T. forsythia* (Fig. 1d).

Based on the demonstrated suitability of GlcNAc-anhMurNAc as a MurNAc source for *T. forsythia* wild-type (Fig. 1d), also the effect of synthesized anhMurNAc at a concentration of 10 μg ml^− 1^ on bacterial growth was investigated. Unexpectedly, also anhMurNAc alone supported growth of *T. forsythia* wild-type, however, it constitutes a rather poor substrate with a reproducibly maximal OD_600_ of ~ 0.32 (Fig. 1a).

### Digestion of PGN by *T. forsythia* cell extracts

**Fig. 3:**
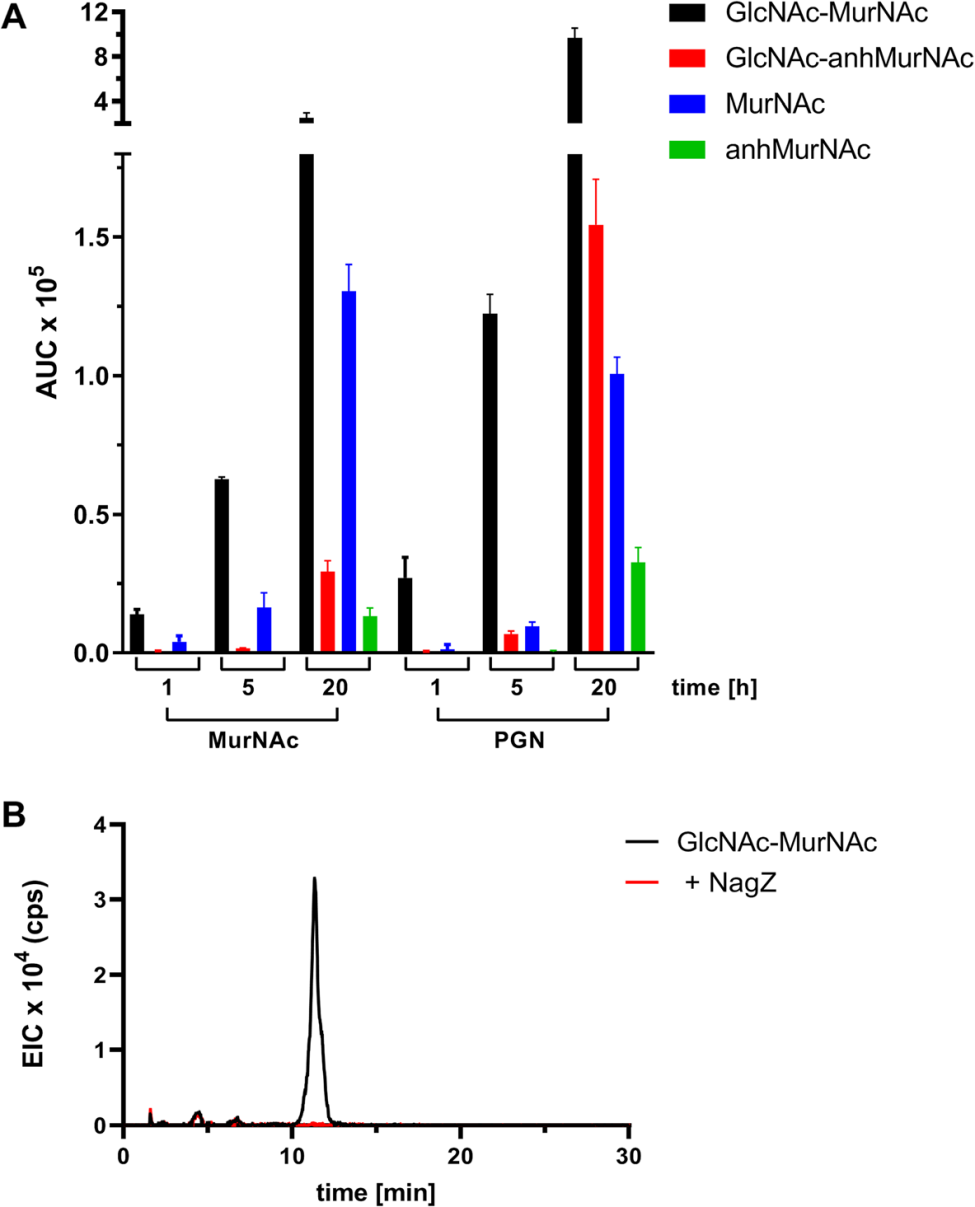
Digestion products obtained upon incubation of *E. coli* PGN with cell extracts of *T. forsythia*, prepared from cultures grown with MurNAc or PGN. Samples were taken after several time points and analysed by LC-ESI-MS in negative ion mode, showing the presence of GlcNAc-MurNAc - *m/z* 495.18 [M-H]^−^, GlcNAc-anhMurNAc - *m/*z 477.17 [M-H]^−^, MurNAc - *m/z* 292.11 [M-H]^−^ and anhMurNAc - *m/z* 274.09 [M-H]^−^. Measured masses were in accordance with the calculated masses of *m/z* 495.18, *m/z* 477.17, *m/z* 292.10, and *m/z* 274.09, respectively. Shown are the mean values of triplicates calculated from the area under the curve (AUC) (**a**). The disaccharide was confirmed to be GlcNAc-MurNAc based on the successful digestion with the *N*-acetylglucosaminidase NagZ, shown as the extracted ion chromatogram (EIC) upon elution from a SeQuant® ZIC®-pHILIC column, before (black) and after (red) enzyme digestion (**b**)

To obtain first insight into the enzymatic activities *T. forsythia* might require for PGN scavenging, a *T. forsythia* cell extract was used as a putative source of such enzymes and digestion products of *E. coli* PGN after treatment with the extract were determined. For this purpose, *T. forsythia* wild-type was cultivated until the late exponential growth phase, either grown with free MurNAc or with *E. coli* PGN as a MurNAc source since we reasoned that PGN-active enzymes might be induced upon growth on PGN. Subsequently, cells were lysed and harvested by ultracentrifugation to prepare enzyme-rich extracts. Finally, a preparation of *E. coli* PGN was incubated with the extract overnight at 37 °C and aliquots were analysed by LC-MS after several time points. We found that a cell extract of *T. forsythia* successfully digested PGN, revealing the disaccharides GlcNAc-MurNAc and GlcNAc-anhMurNAc as well as MurNAc as predominant digestion products in addition to a small amount of anhMurNAc, and, plausibly, peptides of different length (not shown), which all increased over the analysed incubation time of 20 h (Fig. 3a, Supplemental Fig. S5). No difference in digestion products was evident between cultivation of *T. forsythia* with MurNAc *versus* PGN, however, the amount of detected products was higher when using a cell extract prepared from cultures grown with PGN; this was especially true for GlcNAc-MurNAc and GlcNAc-anhMurNAc, which increased by 3.8-fold and 5.3-fold, respectively, with GlcNAc-MurNAc being the major digestion product in absolute number occurring in an 8.6-fold excess compared to GlcNAc-anhMurNAc. GlcNAc-MurNAc was the major disaccharide digestion product obtained regardless of the MurNAc source used for the preparation of the cell extract; this was unambiguously confirmed based on the successful digestion with the *N*-acetylglucosaminidase NagZ [20] (Fig. 3b).

### Role and specificity of *T. forsythia* AmpG in the uptake of MurNAc substitutes

To elucidate the role of the muropeptide transporter AmpG in uptake of MurNAc substitutes, an isogenic mutant of *T. forsythia* was constructed by homologous recombination (*T. forsythia* Δ*ampG*) (Supplemental Fig. S6) and cultivated with the aforementioned MurNAc sources. In comparison to *T. forsythia* wild-type, *T. forsythia* Δ*ampG* suffered from severe growth impairment, when cultivated with alternate MurNAc sources. While *T. forsythia* Δ*ampG* provided with 10 μg ml^− 1^ MurNAc yielded an OD_600_ of 1.5 (Fig. 1a) – supportive of the fact that MurNAc is imported primarily via MurT [23] and not via AmpG - the cultivation with supernatants of the cohabiting bacteria *F. nucleatum* and *P. gingivalis*, as well as *E. coli* Δ*ampG* led to growth inhibition (Fig. 1b). Substitution of MurNAc by undigested PGN enabled restricted growth of *T. forsythia* Δ*ampG* to an OD_600_ of ~ 0.3 (Fig. 1c), indicating that the majority of processed PGN is imported via AmpG; the residual growth might be an effect of free MurNAc, which we showed to be a product of PGN digestion by a *T. forsythia* extract (compare with Fig. 3a). Concomitantly, SEM demonstrated the change of *T. forsythia* Δ*ampG* cells from rod-shaped to fusiform morphology, as is typical under MurNAc starvation (Fig. 2). The supply of specific PGN digests also resulted in growth inhibition of *T. forsythia* Δ*ampG.* Surprisingly, not only a PGN digest with Slt70, containing mainly GlcNAc-anhMurNAc(−peptides) - the preferred substrates of *E. coli* AmpG [22] - led to growth inhibition, but also digests with mutanolysin, containing GlcNAc-MurNAc(−peptides) failed in sustaining growth of *T. forsythia* Δ*ampG* (Fig. 1d).

**Fig. 4:**
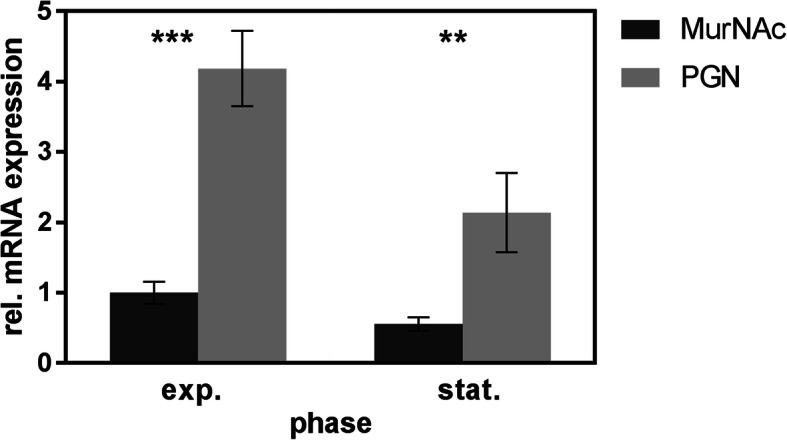
Relative mRNA levels of *ampG* in *T. forsythia* wild-type cultures grown with MurNAc (black) or PGN (grey) until the exponential and stationary growth phase, respectively, as determined by qPCR. Standard errors of triplicates are shown and asterisks indicate significant differences between samples, determined by the unpaired Student’s t-test (**P ≤ 0.01, ***P ≤ 0.001)

Substitution of MurNAc by anhMurNAc restricted growth of *T. forsythia* Δ*ampG* to an OD_600_ of ~ 0.28, which is comparable to the growth of the wild-type upon provision of anhMurNAc, ruling out the involvement of AmpG in the uptake of anhMurNAc (Fig. 1a).

### Determination of relative expression level of AmpG

The direct contribution of the *T. forsythia* inner membrane transporter AmpG (*Tanf_08365*) in the utilization of PGN as a MurNAc source by the pathogen was supported by determining the relative expression level of AmpG under PGN supplementation in comparison to free MurNAc. RNA was isolated from *T. forsythia* wild-type cultivated with either MurNAc or *E. coli* PGN, transcribed into cDNA, and the relative expression level of AmpG was determined by qPCR. When the AmpG expression level in cultures grown with MurNAc until the exponential growth phase was normalized to 1, the relative expression level in the stationary phase was 0.56. In contrast, when cultivated with PGN, the relative expression level was 4.19 in the exponential and 2.14 in the stationary growth phase. In conclusion, when *T. forsythia* was cultivated with PGN, AmpG was upregulated approximately 4.1-fold in the exponential and 3.8-fold in the stationary growth phase (Fig. 4). Further, the data supports a higher AmpG import rate of PGN fragments during the exponential growth phase, which conforms with previous observations made by others in the context of *E. coli* PGN recycling involving the AmpG permease [22].

### PGN fragments in the supernatant of *T. forsythia* Δ*ampG*

**Fig. 5:**
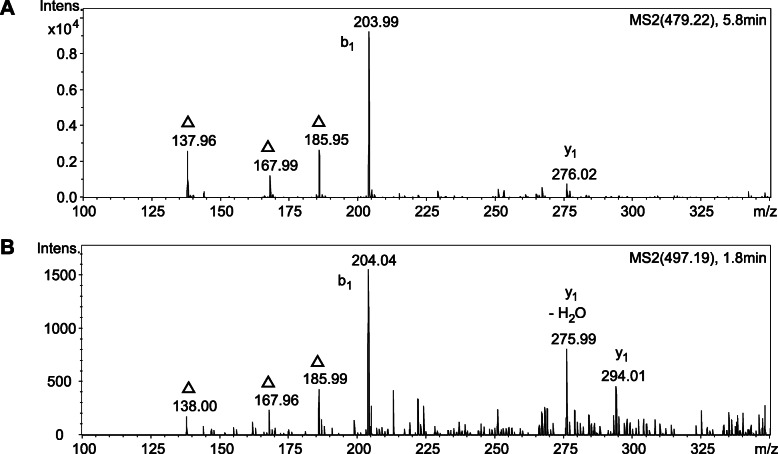
MS^2^ analysis of supernatant from *T. forsythia* Δ*ampG,* showing the presence of GlcNAc-anhMurNAc (**a**) and GlcNAc-MurNAc (**b**)**.** The parent masses of *m/z* 479.22 [M + H]^+^ for GlcNAc-anhMurNAc and *m/z* 497.19 [M + H]^+^ for GlcNAc-MurNAc were in accordance with the theoretical masses of *m/z* 479.19 and *m/z* 497.20, respectively. The b_1_-ion (GlcNAc), the y_1_-ion (MurNAc/anhMurNAc), and the MurNAc signature peaks (denoted by a triangle) support the structural assignment

Since an *E. coli* mutant lacking the inner membrane muropeptide transporter AmpG was shown to release GlcNAc-anhMurNAc into the medium [22], supernatants of *T. forsythia* Δ*ampG* were analysed regarding the accumulation of PGN fragments. *T. forsythia* wild-type and *T. forsythia* Δ*ampG* were cultivated under standard conditions and supernatants were harvested in the stationary growth phase. LC-ESI-MS measurements revealed the presence of the disaccharides GlcNAc-anhMurNAc as well as GlcNAc-MurNAc, only for the mutant *T. forsythia* Δ*ampG*. Observed masses of *m/z* 479.22 [M + H]^+^ for GlcNAc-anhMurNAc and *m/z* 497.19 [M + H]^+^ for GlcNAc-MurNAc were in accordance with the theoretical masses of *m/z* 479.19 and *m/z* 497.20, respectively, and confirmed by MS^2^ analysis (Fig. 5).

## Discussion

Periodontitis is a polymicrobial disease, in which oral biofilm bacteria organize to distribute metabolic activities for increasing their fitness [30]. It is the complexity of bacterial community interactions that is decisive for bacterial dysbiosis and, consequently, the etiology of the disease. This study sought to investigate how the periodontal pathogen *T. forsythia* can benefit from cohabiting biofilm bacteria to overcome its MurNAc auxotrophy*. T. forsythia* possesses a conventional Gram-negative PGN of the A1γ-type [16]; thus, it is conceivable to assume that the bacterium procures the essential PGN building block MurNAc from the oral biofilm consortium. Currently it is unknown, which sources of MurNAc likely available in the oral cavity *T. forsythia* can access. Identifying these sources and understanding how they are imported via the bacterial membrane barriers and metabolized by *T. forsythia* does not only constitute a necessary step for understanding how this pathogen thrives in the oral habitat but can also inform about novel strategies to combat periodontitis, for which no targeted treatment is currently available.

During their life cycle, oral biofilm bacteria - as any kind of bacteria - degrade their PGN cell wall by specific sets of autolytic enzymes and release PGN fragments into the environment; in parallel, they recycle their cell wall turnover products using different strain-specific routes [21]. For *T. forsythia*, recently an inner membrane transporter MurT dedicated to the import of free MurNAc was identified constituting a novel transporter family conserved within the *Bacteroidetes* phylum, to which *T. forsythia* is affiliated [23]. However, it is unclear where the transporter’s MurNAc substrate is derived from. Once imported into the cytoplasm of *T. forsythia*, the path of MurNAc can take two branches. One channels MurNAc into PGN biosynthesis (anabolic route), following conversion into UDP-MurNAc via MurNAc-1P by the subsequent action of the enzymes AmgK and MurU [21] – thereby likely replacing the MurA/B pathway commonly employed by bacteria to generate UDP-MurNAc from UDP-GlcNAc - while the MurK-kinase/MurQ-etherase pathway displays a MurNAc catabolic route generating GlcNAc-6P from MurNAc via MurNAc-6P, which is further shuttled into glycolysis [10]. We have recently shown that deletion of *T. forsythia’s murK* gene blocks MurNAc catabolism and allows the direction of MurNAc to PGN biosynthesis, resulting in a growth advantage of the bacterium in MurNAc-depleted medium [10].

To shed light on *T. forsythia’s* MurNAc sources, we have chosen *P. gingivalis* and *F. nucleatum* as plausible providers of MurNAc sources, both of which are long time-established members of the oral biofilm consortium and known to associate with *T. forsythia* [31]. For comparison, non-oral *E. coli* was included in our study.

Culture supernatants of both *P. gingivalis* and *F. nucleatum* were suitable to sustain growth of *T. forsythia* wild-type (Fig. 1b). Interestingly, GlcNAc-MurNAc was identified as the MurNAc source present in *P. gingivalis,* while GlcNAc-anhMurNAc was found in *F. nucleatum* (Supplemental Fig. S1). This was supported by using the supernatant of an *E. coli* Δ*ampG* mutant that is known to release the GlcNAc-anhMurNAc disaccharide [25]. Noticeably, MS analysis of supernatants was first considered impossible, since the cultivation medium constitutes an extremely complex matrix. Tedious optimisation efforts and application of MS^2^ analysis finally allowed a reproducible detection of small amounts of PGN fragments in the massive noise of supernatants. When comparing maximally reachable OD_600_ values of the *T. forsythia* cultures upon supplying the different bacterial supernatants, it needs to be stressed that while supernatants of the different bacterial cultures were harvested at the same OD_600_ values, slight differences in the final MurNAc concentration applied due to the necessary concentration procedure. In silico analyses showed that the genomes of *P. gingivalis* and *F. nucleatum* lack enzymes dedicated to PGN recycling, such as the anomeric MurNAc/GlcNAc kinase AmgK as well as the anhMurNAc kinase AnmK catalysing the conversion from anhMurNAc into MurNAc-6P. This might explain the release of PGN fragments by these bacteria during cell wall turnover. For comparison, an *E. coli* supernatant which did not contain any detectable MurNAc source due to its efficient PGN recycling, could not sustain growth of *T. forsythia* (Fig. 1b). The utilization of GlcNAc-anhMurNAc as the sole MurNAc source might implicate that *T. forsythia* is able to utilize anhMurNAc as a MurNAc substitute, at least under the applied conditions. To follow up this consideration, growth experiments with anhMurNAc as a single sugar were performed, showing, however, only restricted growth of *T. forsythia* (Fig. 1a). This might be due to inefficient import of anhMurNAc or a low enzymatic metabolization rate of the compound in the cytoplasm. In *E. coli*, anhMurNAc can enter the cytoplasm via the phosphotransferase system-dependent MurNAc transporter MurP, which is absent in *T. forsythia*, where it is further phosphorylated by the anhMurNAc kinase AnmK, as shown by Uehara et al. [32]. On the other hand, the authors reported that *E. coli* could not grow in minimal medium supplemented with anhMurNAc, in contrast to MurNAc, and, thus, assumed that anhMurNAc might be toxic to cells and readily exported by an efflux pump, if not phosphorylated. The contribution of AmpG as an anhMurNAc transporter in *T. forsythia* cells could be ruled out in our study, since growth of *T. forsythia* Δ*ampG* upon provision of anhMurNAc was restricted to a similar level as the wild-type (Fig. 1a). While *T. forsythia’*s MurNAc transporter MurT could be considered as a reasonable transporter for anhMurNAc, the subsequent metabolic strategy remains to be explored, since in the *T. forsythia* genome, no AnmK ortholog for conversion of anhMurNAc into MurNAc-6-phosphate is predicted.

Furthermore, we showed that *T. forsythia* can metabolize intact PGN, regardless of the PGN type (i.e., A1γ in the case of *P. gingivalis* and *E. coli*, and A1δ in the case of *F. nucleatum*) to cover its MurNAc requirements (Fig. 1c). We conclude that *T. forsythia* expresses an active muramidase and a lytic transglycosylase for processing non-native PGN for subsequent recycling, since a *T. forsythia* cell extract yielded GlcNAc-MurNAc, GlcNAc-anhMurNAc as well as MurNAc and anhMurNAc as digestion products from *E. coli* PGN (Fig. 3a, Supplemental Fig. S5). Thus, *T. forsythia* seems to mainly scavenge PGN sugar backbone fragments, most likely generated from muropeptides upon cleavage of peptides by an active amidase, for building up its cell wall PGN; this is supported by the fact that *F. nucleatum* PGN, which has a different stem peptide composition compared to *T. forsythia’s* endogenous PGN, is also growth promoting for the bacterium.

Concerning further processing of PGN fragments in *T. forsythia* cells, the involvement of the AmpG permease *Tanf_08365* in muropeptide uptake was recently shown indirectly in an *E. coli* host, in which AmpG-dependent accumulation of cytosolic muropeptides was evident via a muropeptide inducible β-lactamase reporter gene [11]. Within this present study, we investigated the role of *Tanf_08365* directly in *T. forsythia* by using an isogenic *T. forsythia* Δ*ampG* mutant. Our approach also allowed us to determine the specificity of *T. forsythia* AmpG. Although we cannot rule out that *T. forsythia* AmpG may also transport (anhydro-)muropeptides, our results indicate that GlcNAc-MurNAc and GlcNAc-anhMurNAc disaccharides lacking peptide substituents are the major substrates of the transporter when *T. forsythia* grows on PGN.

AmpG belongs to permeases from the major facilitator superfamily of transport proteins, which are ubiquitously present in Gram-negative bacteria [33–35]. According to the current accumulated data, the principal requirement of AmpG is for the presence of the disaccharide GlcNAc-anhMurNAc. These unique substrates for AmpG, which contain murein peptides linked to GlcNAc-anhMurNAc, are produced by PGN turnover during exponential growth [22].

Our results demonstrated that *Tanf_08365* is essential for growth of *T. forsythia* on complex MurNAc sources provided by other bacteria. This was supported by SEM analysis of cell morphology (Fig. 2). Growth of *T. forsythia* Δ*ampG* was severely impaired when cultivated with intact PGN of cohabiting bacteria (*i.e., P. gingivalis* or *F. nucleatum*) and even completely inhibited when cultivated with supernatants (Fig. 1b, c). In comparison, upon supplementation of free MurNAc, which can enter the cytoplasm via the inner membrane transporter MurT [23], growth of *T. forsythia* Δ*ampG* until maximally reachable OD_600_ was observed (Fig. 1a). Supportive of this finding, we found that the relative expression level of AmpG was significantly upregulated when *T. forsythia* was cultivated with PGN instead of free MurNAc (Fig. 4).

Considering that an *E. coli* PGN digest by the *T. forsythia* cell extract yielded GlcNAc-MurNAc, besides the classical PGN recycling product GlcNAc-anhMurNAc, it was conceivable to assume that both these disaccharides would be substrates of *T. forsythia* AmpG. Thus, we produced defined fragments of *E. coli* PGN by controlled enzymatic digestion and provided these as MurNAc substitutes for growth of *T. forsythia* wild-type versus Δ*ampG*. While growth of the wild-type was unrestrictedly sustained when cultivated with GlcNAc-MurNAc(−peptides) and only slightly growth-impaired with GlcNAc-anhMurNAc(−peptides), *T. forsythia* Δ*ampG* was completely growth inhibited in either case (Fig. 1d). Additionally, both disaccharides - GlcNAc-anhMurNAc and GlcNAc-MurNAc - were detected in culture supernatants of *T. forsythia* Δ*ampG* (Fig. 5), comparable to the situation found in *E. coli* Δ*ampG*, that, however, releases only GlcNAc-anhMurNAc. Regarding the importance of peptides being attached to the disaccharides, no difference in growth of *T. forsythia* wild-type was observed when cultivated with disaccharide-peptides or disaccharides alone. However, the fact that *T. forsythia* cell extracts exclusively produce disaccharide digestion products and *T. forsythia* Δ*ampG* releases only disaccharides into the medium, might indicate the presence of a strong amidase activity in the periplasm of *T. forsythia*. That *T. forsythia* AmpG is also able to take up disaccharides carrying stem peptides might be concluded from the study by Ruscitto et al. [11], where the cytosolic accumulation of anhMurNAc-peptides in an *E. coli* double mutant lacking the muropeptide transporter AmpG and the amidase AmpD in comparison to the *E. coli* strain complemented with *T. forsythia ampG* indicated that AmpG transported *E. coli*’s main PGN recycling product GlcNAc-anhMurNAc-peptides into the cytoplasm. The usability of muropeptides as a growth factor by *T. forsythia* was supported by the authors of that study by demonstrating that a PGN digest of *F. nucleatum* could sustain bacterial growth. However, the exact composition of these muropeptides remained elusive since the interpretation of the corresponding MS data was based on the presence of *m*-Dap, which, however, is not a constituent of *F. nucleatum* PGN [17].

## Conclusion

The inner membrane transporter AmpG of the periodontal pathogen *T. forsythia* is essential for growth of the bacterium on complex MurNAc sources provided by cohabiting bacteria in the oral cavity and imports both the long-standing substrate GlcNAc-anhMurNAc as well as GlcNAc-MurNAc into the cytoplasm. The capacity or need for also metabolizing GlcNAc-MurNAc(−peptides) might be explained by *T. forsythia’s* MurNAc-auxotrophy. An important question that is currently addressed in our laboratories concerns how the PGN fragments that are imported via AmpG are processed in *T. forsythia.*

This study increases our understanding of the PGN metabolism of *T. forsythia* and simultaneously might reveal a new perspective on AmpG permeases, for which the GlcNAc-1,6-anhydroMurNAc disaccharide has been regarded as a minimum substrate signature in many Gram-negative bacteria. The new finding of this study of also GlcNAc-MurNAc being a growth factor of *T. forsythia* and AmpG substrate might have future implications for AmpG as a potential target for resistance-attenuating therapeutics [36].

## Methods

### Bacterial strains, growth conditions and growth curves

*Tannerella forsythia* ATCC 43037 type strain (*T. forsythia* wild-type), *T. forsythia* ATCC 43037 *∆ampG* (*Tf_ampG::ermF;* for the construction of the mutant see next paragraph), *Fusobacterium nucleatum* subsp*. nucleatum* OMZ 598, and *Porphyromonas gingivalis* W83 were cultivated anaerobically at 37 °C in brain heart infusion medium (BHI; 37 g l^− 1^; Oxoid, Basingstoke, United Kingdom), supplemented with yeast extract (10 g l^− 1^; Sigma, Vienna, Austria), L-cysteine (1 g l^− 1^; Sigma), hemin (5 μg ml^− 1^; Sigma), and menadione (2 μg ml^− 1^; Sigma). For *T. forsythia* strains, 5% (v v^− 1^) horse serum (Thermo Fisher Scientific, Vienna, Austria) and MurNAc (10 μg ml^− 1^ or 1 μg ml^− 1^) Carbosynth, Compton, United Kingdom) were added, if not stated otherwise; *T. forsythia* ATCC 43037 *∆ampG* was grown with additional supplementation of 5 μg ml^− 1^ erythromycin. *Escherichia coli* MC4100 and the *E. coli* TP72 (F^−^ *lysA opp araD139 rpsL150 relA1 deoC1 ptsF25 ftbB5301 rbsR* _(*argF-lac*) *ampG*::Kan) [37] were routinely cultivated in Miller’s LB broth base supplemented with 50 μg ml^− 1^ kanamycin (Thermo Fisher Scientific) at 37 °C with constant shaking. For the preparation of MurNAc substitutes from *E. coli*, BHI medium was used instead of Miller’s LB broth base for comparability.

For recording of growth curves, 10 ml of bacterial cultures were inoculated to a starting optical density at 600 nm (OD_600_) of 0.05 and biological triplicates were measured in 1-ml cuvettes with a cell density meter (Ultraspec 10; Amersham Biosciences, Austria) at several time points until the stationary phase was reached. Samples of OD_600_ < 0.7 were measured directly, while samples of OD_600_ > 0.7, were diluted by 1:10.

### Construction of a *T. forsythia* Δ*ampG* deletion mutant

The gene encoding the AmpG muropeptide transporter homologue (*Tanf_08365*) was deleted by homologous recombination using a knock-out vector, replacing the *ampG* gene by an erythromycin resistance marker gene (Supplemental Fig. S6). A detailed description of the construction of a deletion mutant in *T. forsythia* is published elsewhere [38]. Briefly, a knock-out cassette was constructed by PCR amplification of 1 kbp-long homologous up- and downstream regions of the *ampG* gene, flanking the *ermF* marker gene, using Phusion High-Fidelity DNA polymerase (Thermo Fisher Scientific). Up- and downstream homologous regions were amplified from *T. forsythia* genomic DNA by primer pairs 634/635 and 636/637, respectively, and the *ermF* gene was amplified from vector pJET/dTF0955ko [38] using primer pair 460/461 [39] (Table 1). The final knock-out cassette was produced by overlap extension PCR and blunt-end cloned into the cloning vector pJET1.2 (Thermo Fisher Scientific). Approximately 5 μg of the final knock-out vector pJET1.2/Δ*Tanf_08365* were transformed into 100 μl of *T. forsythia* cells by electroporation and viable clones were selected on BHI agar plates containing erythromycin (5 μg ml^− 1^). The correct integration of the knock-out cassette was tested by screening PCR using primer pairs 638/524 and 525/639 (Supplemental Fig. S6, Table 1).

**Table 1:**
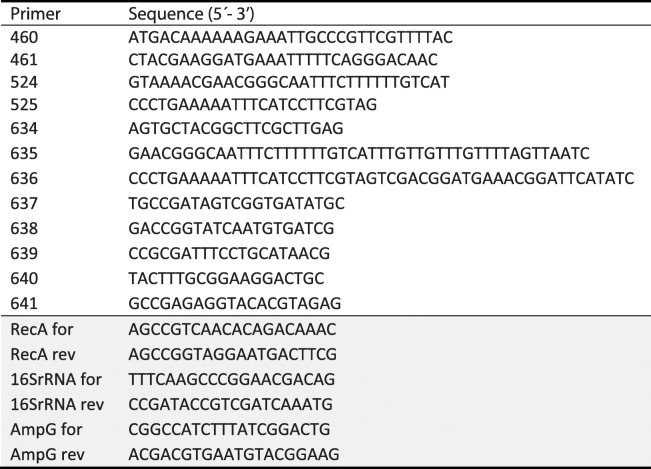
Oligonucleotide primers used for PCR amplification reactions. Primers for qPCR are shaded in grey

### Preparation of MurNAc sources

Three types of MurNAc substitutes were prepared, including bacterial culture supernatants, intact PGN sacculi, and defined PGN fragments.

Culture supernatants were harvested by centrifugation (8000 g, 10 min) from 50 ml of *P. gingivalis, F. nucleatum*, and *E. coli* MC4100 and TP72 (Δ*ampG*) cultures at OD_600_ ~ 2.0 (corresponding to the exponential growth phase), sterile-filtered, and concentrated seven-fold, using a SpeedVac vacuum centrifuge (Thermo Fisher Scientific). For growth experiments, 1 ml of concentrated supernatant, each, was added to 10 ml of *T. forsythia* culture.

PGN sacculi from *T. forsythia*, *P. gingivalis, F. nucleatum*, and *E. coli* were isolated following a published protocol [16, 26]. In short, biomass was harvested from bacterial cultures from the stationary growth phase and boiled in 8% SDS to lyse cells, followed by several washing steps with distilled water using ultracentrifugation (60,000 g, 30 min, 60 °C) and dialysis against distilled water. Subsequently, PGN preparations were treated with α-amylase (24 mg ml^− 1^; Sigma) for 2 h at 37 °C and, subsequently, with pronase (10 mg ml^− 1^; Sigma) for 2 h at 60 °C to degrade contaminating carbohydrates and proteins, boiled, washed, and dried in a SpeedVac vacuum centrifuge [16].

To correct for differences in purity, the MurNAc concentration in the different bacterial culture supernatants and PGN preparations was determined by HPAEC-PAD. For this purpose, seven-fold concentrated bacterial culture supernatants and 400 μg of PGN, respectively, were hydrolysed with 400 μl of 6 N HCl containing 0.2% thioglycolic acid and incubated at 110 °C over-night. Samples were dried, washed, resuspended in 120 μl of distilled water and sterile-filtered, before 20 μl of material were injected into the HPAEC-PAD system ICS3000, using a CarboPac PA1 column (Dionex, Thermo Fisher Scientific) and applying a flow rate of 1 ml min^− 1^. Separation of sugar constituents was performed starting with an isocratic elution of 15% solvent A (100 mM sodium hydroxide in distilled water) and 0.2% solvent B (1 M sodium acetate in solvent A) for 21 min, followed by linear gradients to 98.8% A and 0.2% B until min 27, changing to 68% A and 32% B until min 59, back to 98.8% A and 0.2% B until min 65 and to 15% A and 0.2% B until min 68, maintained until min 80. For growth experiments, PGN isolates were added as a medium component, equalling a MurNAc concentration of ~ 1 μg ml^− 1^.

To obtain defined PGN fragments, 3 mg of *E. coli* PGN was digested with *Streptomyces globisporus N*-acetylmuramidase mutanolysin (50 μg ml^− 1^; Sigma) or *E. coli* lytic transglycosylase Slt70 (100 μg ml^− 1^) [40, 41], dissolved in 10 mM HEPES buffer containing 0.1 M NaCl and 0.1% Triton™ X-100 (Sigma), in a final volume of 420 μl. For digestion with *E. coli* amidase AmiD (50 μg ml^− 1^) [25], 5 μM zinc chloride (Sigma) was added. Incubation was performed at 37 °C under constant shaking overnight and reactions were stopped by heating at 100 °C for 25 min. These specific digests were used as MurNAc substitutes, applying the same concentrations as for undigested PGN.

Slt70 and AmiD were produced as recombinant, His_6_-tagged enzymes in *E. coli* BL21 (DE3) cells carrying the expression plasmid pET29b-slt70 and pET28a-Ec_amiD, respectively. Transformed *E. coli* was grown in 400 ml of LB broth supplemented with kanamycin (50 μg ml^− 1^) at 37 °C and 200 rpm. At the mid-exponential growth phase (OD_600_ ~ 0.6), protein expression was induced with 1 mM isopropyl-β-D-thiogalactopyranosid (IPTG) and cultivation was continued for 4 h. Cells were harvested by centrifugation (4500 g, 20 min, 4 °C). Purification of His_6_-tagged enzymes followed a previously established protocol [34]. Briefly, cell pellets were resuspended in lysis buffer (50 mM Tris-HCl, pH 7.6, 300 mM NaCl) containing 10 mM imidazole and EDTA-free protease inhibitor mixture (cOmplete, Roche Applied Science), followed by ultrasonication. Lysates were centrifuged (40,000 g, 40 min, 4 °C) and the supernatant fraction was applied to a nickel–nitrilotriacetic acid (Qiagen) affinity chromatography column equilibrated in lysis buffer. After washing the column with 10 column volumes of washing buffer (50 mM Tris-HCl, pH 7.6, 300 mM NaCl, 30 mM imidazole), the His_6_-tagged protein was eluted with 200 mM imidazole. Fractions containing the purified enzyme, as determined by SDS-PAGE (12% gel) upon Coomassie Brilliant Blue G250 (CBB) staining, were pooled and dialyzed against dialysis buffer (50 mM Tris-HCl, pH 7.6, 100 mM NaCl) overnight at 4 °C. Protein concentration was measured spectrophotometrically and calculated using A_280_ 0.1% values obtained from the exPASy ProtParam tool (http://web.expasy.org/protparam).

### Analysis of culture supernatants

One hundred μl of bacterial culture supernatants from OD_600_ ~ 2.0-cultures (as described above) were harvested by centrifugation (8000 g, 15 min) and prefiltered over a Durapore™ 0.22 μm cartridge (Merck, Vienna, Austria). Subsequently, samples were dried in a SpeedVac vacuum centrifuge (Thermo Fisher Scientific, Vienna, Austria) and dissolved in 15 μl of 80 mM formate, buffered to pH 3.5 with ammonia (solvent A). In the case of *P. gingivalis*, the supernatant was enriched via HILIC-SPE (2.5 g microcrystalline cellulose; Merck), bringing 100 μl of supernatant to a total volume of 2 ml, matching the starting conditions of the self-packed column (90% acetonitrile in 0.1% trifluoroacetic acid (TFA)), followed by washing with 10 ml 90% acetonitrile in 0.1% TFA and elution with 5 ml of 40% acetonitrile in 0.1% TFA. Separation was performed with a Kinetex XB-C18 column (150 × 2.1 mm, 100 Å, 1.7 μm; Phenomenex) on a Nexera X2 HPLC (Shimadzu, Korneuburg, Austria) connected to an amaZon ion trap mass spectrometer (Bruker, Bremen, Germany). At a flow rate of 0.25 ml min^− 1^ and a column oven temperature of 42 °C, an initial concentration of 2% solvent B (80% acetonitrile in solvent A) was held for 5 min before applying a linear gradient to 10% B over 15 min. Percentage B was further raised to 35 and 95% over 5 and 15 min, respectively. Operation of the ion trap was done in the positive ion mode with DDA (Capillary 4.5 kV). Data was analysed in PostrunAnalysis with LabSolutions 5.73 (Shimadzu, Germany) and Bruker Daltonics DataAnalysis 4.0.

### Analysis of peptidoglycan fragments

PGN fragments produced by digestion of *E. coli* PGN with mutanolysin, Slt70, and AmiD, respectively, were analysed by LC-ESI-MS, as described above under “Analysis of culture supernatants”.

PGN-type analysis of *F. nucleatum* was performed following a recently published protocol [16]. Briefly, PGN was resuspended in 200 mM sodium phosphate buffer, pH 6.0, digested with *S. globisporus* mutanolysin (50 μg ml^− 1^; Sigma) overnight at 37 °C and reduced with sodium borohydride. The supernatant was dried in a SpeedVac vacuum centrifuge (Thermo Fisher Scientific) and preparations were applied to HPLC, as published elsewhere [42]. LC-ESI-MS measurements were performed using a C18 Gemini column (150 × 4.6 mm, 110 Å, 5 μm; Phenomenex) and an UltiMate 3000 HPLC system (Dionex) coupled to a MicrO-TOF II mass spectrometer (Bruker), operated in positive ion mode [42].

### Synthesis of anhydro-*N*-acetylmuramic acid

To serve as a standard for the analysis of the different MurNAc sources investigated within the frame of this study and as a putative growth supplement for *T. forsythia*, anhMurNAc was chemically synthesized via a five-step synthesis scheme according to a published protocol [29], with minor modifications. For the introduction of the trityl group at position four during the third step, the formation of the reagent trityl triflate beforehand is necessary. This step revealed to be very temperature sensitive and only worked, if the addition of tritylalcohol to a solution of trimethylsilyl trifluoromethanesulfonate was carried out at low temperature (− 78 °C). Once trityl triflate had been formed, it could be used for the introduction of the trityl group also at moderate temperatures (0 °C).

The purification of the final deprotected anhMurNAc was achieved by HILIC. Crude deprotected anhMurNAc was dissolved in a minimal amount of water and injected via a sample loop into a semi-preparative HILIC column (SeQuant ZIC HILIC column -Merck, 25 × 1 cm, Cat No 1.50494.0001) connected to an Interchim 4125 ELSD preparative HPLC system. A gradient from 100% acetonitrile to 40% acetonitrile and 60% 5 mM ammonium acetate over 10 column volumes was employed. Elution was monitored by ELSD, product-containing fractions were collected, dried in a SpeedVac centrifuge and checked individually by NMR spectroscopy.

NMR spectra were recorded at 297 K in 99.9% acetonitrile with a Bruker Avance III 600 spectrometer (^1^H at 600.13 MHz, ^13^C at 150.9 MHz), using standard Bruker NMR software. ^1^H NMR were referenced internally to the residual solvent signal (1.94 ppm). Care needed to be taken upon analysing anhMurNAc by NMR, as only the protonated form is soluble in acetonitrile, for which the shifts have been reported [29] and could be reproduced. The chemical shifts in other solvents such as MeOD differed greatly, even from fraction to fraction (this study). Therefore, fractions after HILIC purification were acidified to a pH of 4.0 (Dowex 50XW8) before further processing.

In growth experiments, anhMurNAc was added at a concentration of 10 μg ml^− 1^ to substitute MurNAc.

### Digestion of peptidoglycan by *T. forsythia* cell extracts

To determine an overall PGN degrading activity of soluble *T. forsythia* enzymes, three biological replicates of *T. forsythia* wild-type supplemented with free MurNAc (10 μg ml^− 1^) or *E. coli* PGN were cultivated as described above. When an OD_600_ of 0.8 had been reached, 30 ml of *T. forsythia* culture were harvested by centrifugation (8000 g, 10 min, 4 °C). Cells were washed, resuspended in 1.5 ml of 10 mM Tris-HCl, pH 8.0, and lysed by ultrasonication (4 × 30 s, on ice). After centrifugation (14,000 g, 15 min, 4 °C), the enzyme-rich supernatant was incubated with 0.5 mg of *E. coli* PGN at 37 °C. Samples were prepared after 0, 1, 5 and 20 h by mixing 300 μl of incubation solution with 1.2 ml of ice-cold acetone followed by incubation for 15 min at 4 °C. After centrifugation (12,000 g, 10 min, 4 °C), the supernatant was dried in a SpeedVac vacuum centrifuge (Thermo Fisher Scientific) and resuspended in 100 μl of distilled water. Five-microliters of each sample were analysed by LC-ESI-MS operated in negative ion mode, applying a 45-min HPLC program (for 5 min, 100% buffer A: 0.1% formic acid with 0.05% ammonium formate, then 30 min of a linear gradient to 40% buffer B (CH_3_CN), and 10 min of 100% buffer A for column re-equilibration) according to [43], including an optimized column re-equilibration step. Exact masses for MurNAc, GlcNAc-MurNAc, GlcNAc-anhMurNAc and anhMurNAc, were presented as extracted ion chromatograms using Data Analysis 4 (Bruker), and the peak areas under the curve were quantified with GraphPad Prism 6.

To analyse disaccharides consisting of GlcNAc and MurNAc, samples were further digested with 50 μg ml^− 1^ of *Bacillus subtilis N*-acetylglucosaminidase NagZ [43] (available in the laboratory of C. Mayer) for 4 h at 37 °C. Five-microliters of each sample were analysed immediately by LS-MS run in negative ion mode as described previously [44] in order to ensure the efficient separation of GlcNAc-MurNAc and MurNAc on a SeQuant® ZIC®-pHILIC column (Merck, PEEK 150 × 2.1 mm, 5 μm).

### Determination of the relative expression level of AmpG by qPCR

To determine, if *ampG* was upregulated upon growth with PGN as a MurNAc source, three biological replicates of *T. forsythia* wild-type were cultivated upon supplementation with *E. coli* PGN (corresponding to a MurNAc concentration of 1 μg ml^− 1^) and, for comparison, free MurNAc, as described above, and 10 ml of culture from the exponential phase and 5 ml of culture from the stationary phase, respectively, were harvested by centrifugation (8000 g, 10 min). RNA was isolated using PureLink™ RNA Mini Kit (Thermo Fisher Scientific) with TRIzol™ reagent as described by the manufacturer, including an on-column PureLink™ DNAase (Thermo Fisher Scientific) treatment. Fifty ng of RNA were transcribed to cDNA, using the High-Capacity cDNA Reverse Transcription Kit (Thermo Fisher Scientific). For each cDNA sample, a reverse transcriptase control was included to test for genomic DNA contaminations. Standards were prepared for the target gene *ampG* and the house-keeping genes *recA* and 16SrRNA by PCR, using the Phusion High-Fidelity DNA Polymerase (Thermo Fisher Scientific), listed primers (Table 1), and genomic DNA of *T. forsythia* isolated according to a published protocol [45]. After verifying the specificity of primers by agarose gel electrophoresis (1.5%, 80 V, 40 min), PCR products were purified using the GeneJET Gel Extraction Kit (Thermo Fisher Scientific) and diluted in a series of 1:10-steps to 10^8^–10^3^ copies per μl for generating a standard curve. Quantitative real-time PCR was performed with gene specific primers (Table 1) on a Rotor-Gene Q real-time PCR cycler (Corbett Research, Qiagen, Germantown, MD, USA), using Power SYBR Green PCR Master Mix (Thermo Fisher Scientific). Reactions were set up in triplicate, including 5 μl master mix, 1 μl primer mix (500 nM), 3 μl DEPC-treated water (Thermo Fisher Scientific), and 1 μl cDNA. Relative expression levels of *ampG* were quantified using the relative standard curve method and normalized to the expression of house-keeping genes *recA* and 16S rRNA, using the geometric mean as described by Taylor et al. [46]. Statistically significant differences (*P* < 0.05) between groups were determined using the unpaired student’s t-test.

### Scanning electron microscopy (SEM)

*T. forsythia* wild-type and *T. forsythia ∆ampG* were cultivated with free MurNAc or *E. coli* PGN, as described above, and samples were prepared for SEM following a published protocol [38]. In short, 1 ml of each culture was harvested by centrifugation (8000 g, 7 min), cell pellets were washed with phosphate buffered saline and applied to increasing concentrations of ethanol from 25 to 100% in PBS, with incubation of 7 min and removal of ethanol by centrifugation after each step. Samples were sputter-coated with gold (EM SDC005 apparatus; Leica, Wetzlar, Germany) and imaged with an Inspect S50 scanning electron microscope (FEI, Eindhoven, The Netherlands).

## Supplementary information

**Additional file 1.**

## Data Availability

The datasets used and/or analysed during the current study are available from the corresponding author on reasonable request.

## Abbreviations

Ala: Alanine
anhMurNAc: anhydro-*N*-acetylmuramic acid
*m*-DAP: *meso*-diaminopimelic acid
*E. coli*: *Escherichia coli*
ELSD: Evaporative light-scattering detection
*F. nucleatum*: *Fusobacterium nucleatum*
GlcNAc: *N*-acetylglucosamine
Glu: Glutamic acid
HILIC: Hydrophobic interaction liquid chromatography
HPAEC-PAD: High-performance anion exchange chromatography with pulsed amperometric detection
IPTG: Isopropyl-β-D-thiogalactopyranosid
LC-ESI-MS: Liquid chromatography-electrospray ionization-mass spectrometry
MeDO: Deuterated methanol
MS^2^: Tandem mass spectrometry
MurNAc: *N*-acetylmuramic acid
NMR: Nuclear magnetic resonance
*P. gingivalis*: *Porphyromonas gingivalis*
PGN: Peptidoglycan
qPCR: quantitative real-time PCR
RP-HPLC: Reversed phase high-performance liquid chromatography
*S. globisporus*: *Streptomyces globisporus*
SPE: Solid phase extraction
TFA: Trifluoroacetic acid
*T. forsythia*: *Tannerella forsythia*
