# Supplementary material for: Utilization of different MurNAc sources by the oral pathogen Tannerella forsythia and role of the inner membrane transporter AmpG
Source: BMC Microbiol. 2020 Nov 17;20:352. doi: 10.1186/s12866-020-02006-z (PMC7670621; doi:10.1186/s12866-020-02006-z)
Supplement: Supplementary file 1 — Additional file 1. [file 12866_2020_2006_MOESM1_ESM.docx]

***Supplemental Figures***

**Utilization of different MurNAc-sources by the oral pathogen *Tannerella forsythia* and role of the inner membrane transporter AmpG**

**Valentina M. T. Mayer^1^, Markus B. Tomek^1^_,_ Rudolf Figl^2^, Marina Borisova^3^, Isabel Hottmann^3^, Markus Blaukopf^4^, Friedrich Altmann^2^, Christoph Mayer^3*^ and Christina Schäffer^1*^**

^1^Department of NanoBiotechnology, *NanoGlycobiology* unit, Universität für Bodenkultur Wien, Vienna, Austria

^2^Department of Chemistry, Institute of Biochemistry, Universität für Bodenkultur Wien, Vienna, Austria

^3^Microbiology/Glycobiology, Interfaculty Institute of Microbiology and Infection Medicine Tübingen, Department of Biology, Eberhard Karls Universität Tübingen, Tübingen, Germany

^4^Department of Chemistry, Institute of Organic Chemistry, Universität für Bodenkultur Wien, Vienna, Austria

*Correspondence: [christina.schaeffer@boku.ac.at](mailto:christina.schaeffer@boku.ac.at); [christoph.mayer@uni-tuebingen.de](mailto:christoph.mayer@uni-tuebingen.de)

**
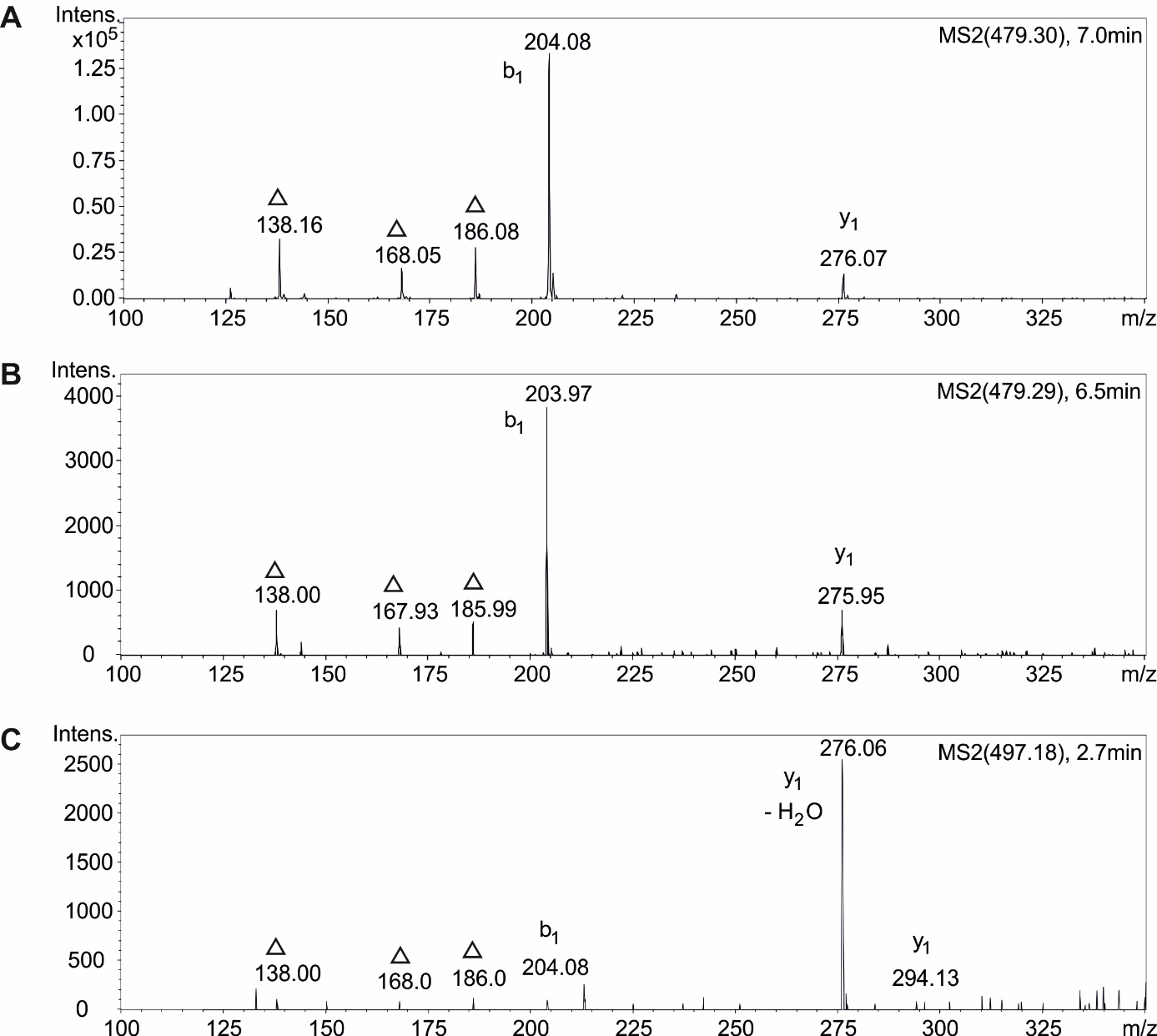
**

**Fig. S1** MS^2^ analysis of supernatants of *E. coli* Δ*ampG* **(A)** and *F. nucleatum* **(B)**, showing the presence of GlcNAc-anhMurNAc, and of *P. gingivalis* **(C)**, showing the presence of GlcNAc-MurNAc. Conforming with the theoretical mass of *m/z* 479.19 for GlcNAc-anhMurNAc and *m/z* 497.19 for GlcNAc-MurNAc*,* the disaccharides were observed with a parent mass of *m/z*479.30 **(A)**, *m/z* 479.29 **(B)** and *m/z* 497.18 [M+H]^+^ **(C)**, respectively. The b_1_-ion (GlcNAc), the y_1_-ion (MurNAc/anhMurNAc) and the MurNAc signature peaks (denoted by a triangle) support the structural assignment.


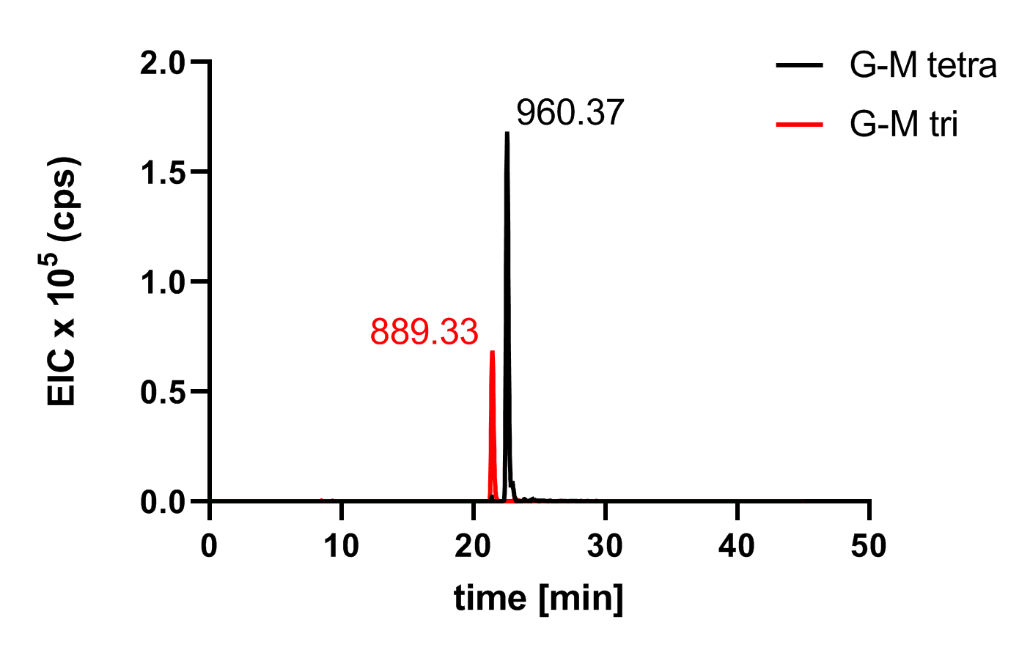


**Fig. S2** LC-ESI-MS analysis of *F. nucleatum* PGN digests with mutanolysin yielding GlcNAc-MurNAc-peptides, containing of GlcNAc (G), MurNAc (M), alanine, glutamic acid, lanthionine (tri) and alanine (tetra), showing the extracted ion chromatogram (EIC) upon elution from a C18 Gemini column. Conforming with the theoretical masses in a reduced state of *m/z* 889.33 and *m/z*960.37, observed peaks were *m/z*889.32 [M+H]^+^ for G-M-tri and *m/z* 960.36 [M+H]^+^ for G-M-tetra.

*
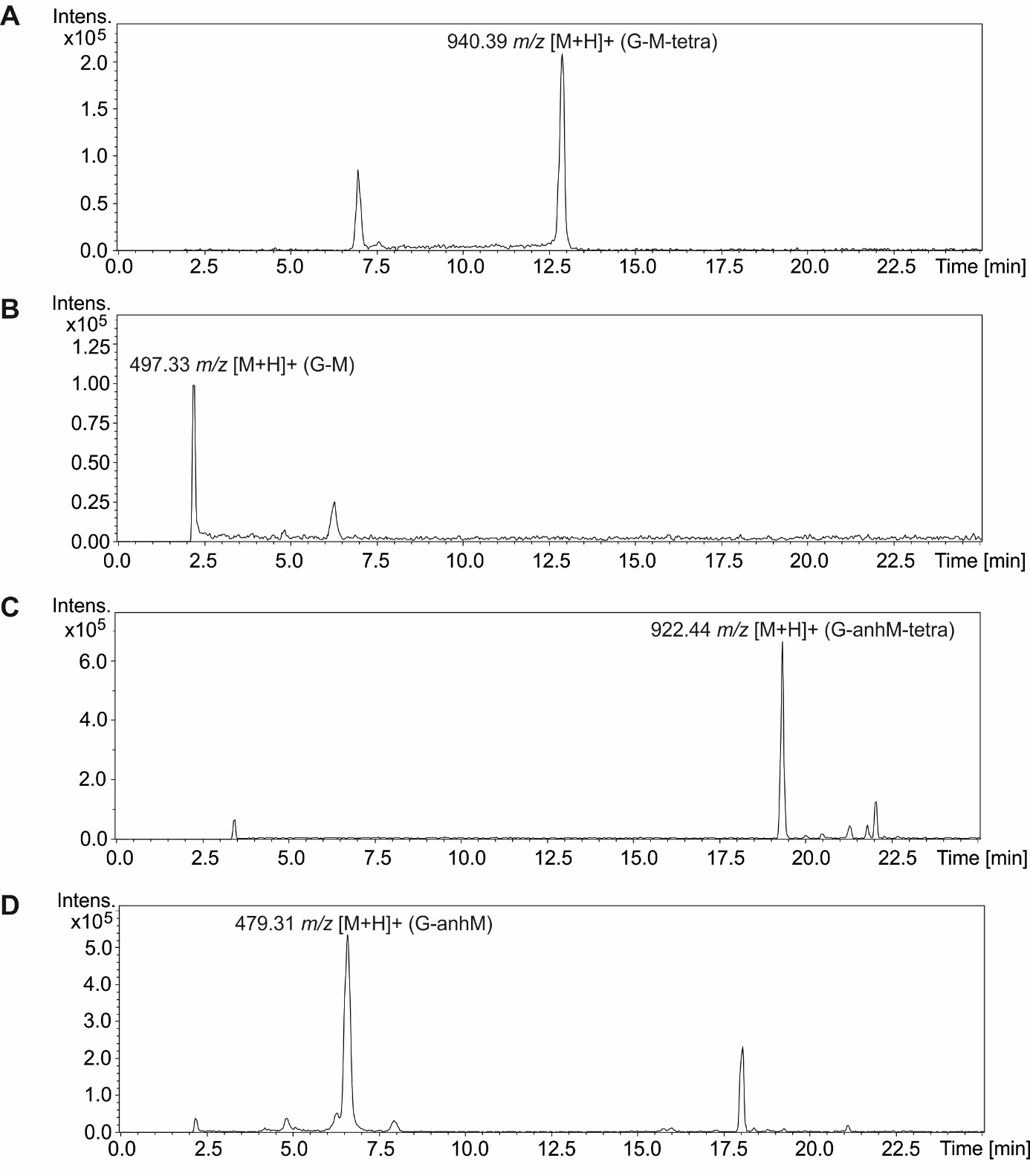
*

**Fig. S3** LC-ESI-MS analysis of specific digests of *E. coli* PGN, showing the base peak chromatograms of representative digestion products, including GlcNAc-MurNAc-tetra (G-M-tetra) for digestion with mutanolysin **(A)**, GlcNAc-MurNAc (G-M) for the combined digestion with mutanolysin and AmiD **(B)**, GlcNAc-anhMurNAc-tetra (G-anhM-tetra) for digestion with Slt70 **(C)** and GlcNAc-anhMurNAc (G-anhM) for the combined digestion with Slt70 and AmiD **(D)**.


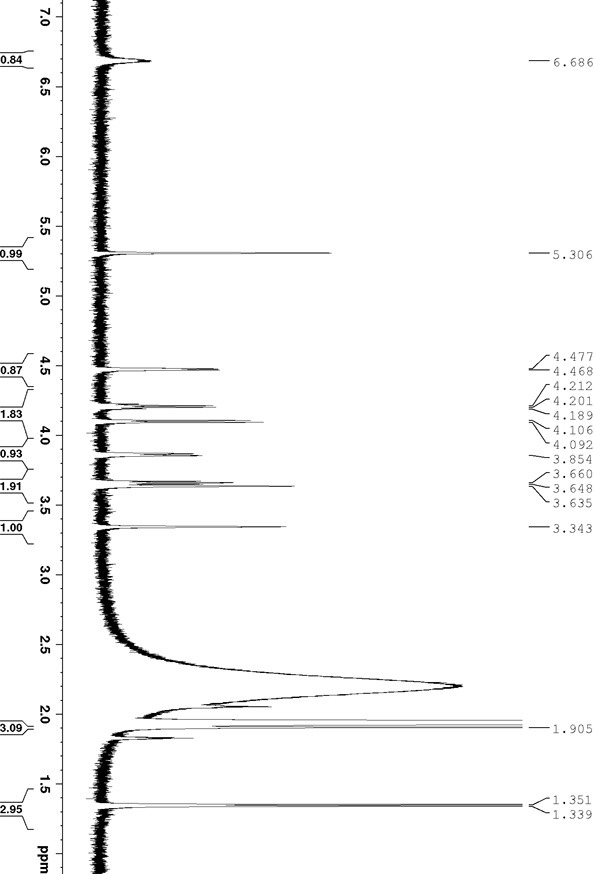


**Fig. S4** ^1^H NMR spectrum (600.13 MHz) of 1,6-anhydro-*N*-acetylmuramic acid.


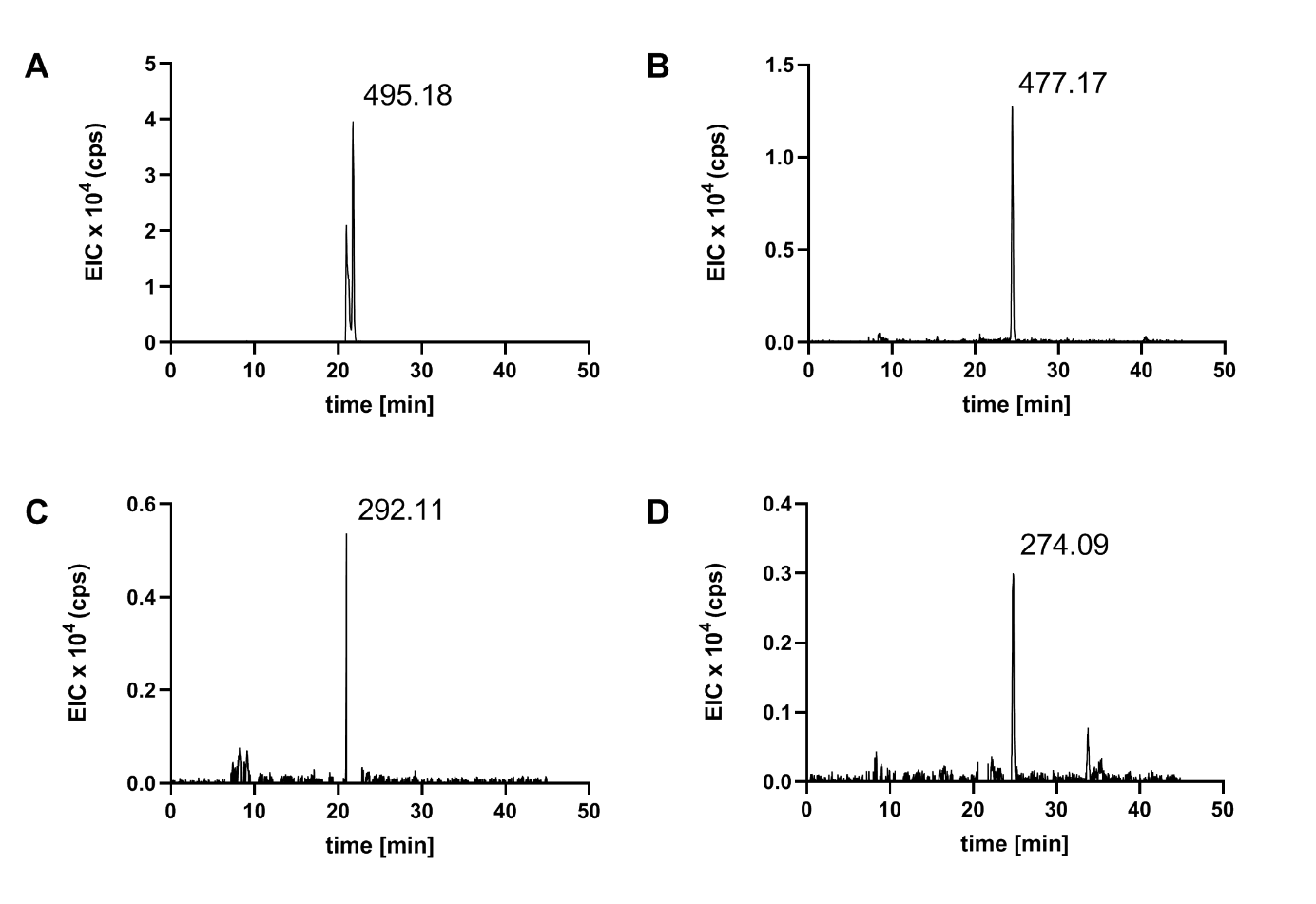


**Fig. S5** LC-ESI-MS analysis of digestion products obtained upon incubation of *E. coli* PGN with cell extracts of *T. forsythia*, as presented in Fig. 4 A. Extracted ion chromatograms (EIC) upon elution from a C18 Gemini column are exemplarily shown for one of each measurement of extracts prepared from *T. forsythia* cultivated with PGN and incubated with *E. coli* PGN for 20 hours. Measured masses of GlcNAc-MurNAc ‑ *m/z*495.18 [M-H]^-^ **(A)**, GlcNAc-anhMurNAc ‑ *m/*z 477.17 [M-H]^-^ **(B),** MurNAc ‑ *m/z* 292.11 [M-H]^-^ **(C)** and anhMurNAc ‑ *m/z*274.09 [M-H]^-^ **(D)** were in accordance with the calculated masses of *m/z*495.18, *m/z* 477.17, *m/z*292.10, and *m/z* 274.09, respectively.


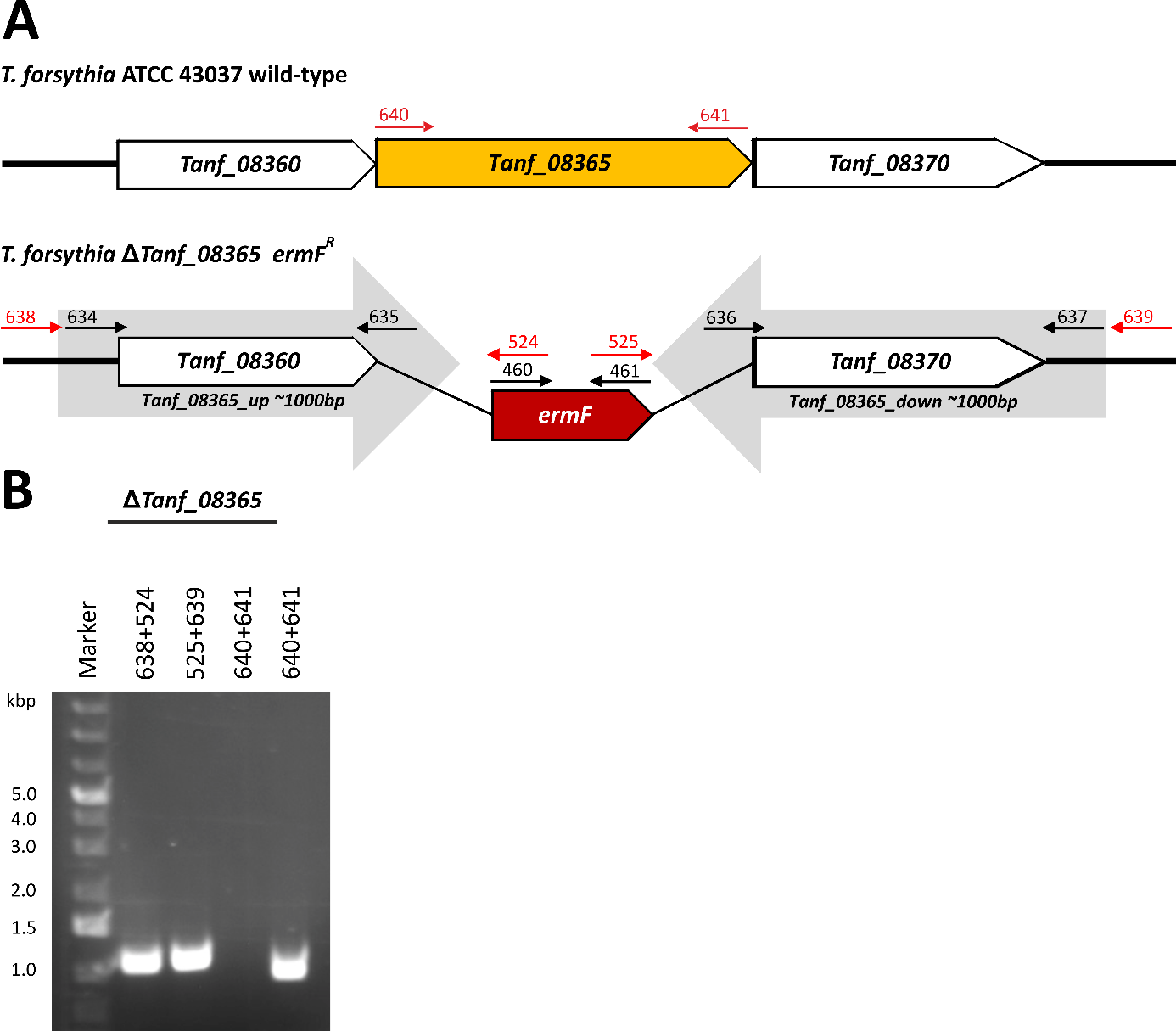


**Fig. S6** Strategy for the construction of a *T. forsythia* ATCC 43037 *ampG* (*Tanf_08365*) deficient mutant and confirmation by PCR. **(A)** The genomic organization of the *Tanf_08365* locus is shown for the parent strain *T. forsythia* ATCC 43037 and the Δ*Tanf_08365* mutant. Black coloured arrows represent primers used for PCR amplification of genes and homologous regions, red coloured primers represent those used to screen for correct integration of the knock-out cassette (not drawn to scale). **(B)** Agarose gel electrophoresis confirms the deletion of *Tanf_08365* using the up-stream primers 638/524 (1066 bp) and down-stream primers 525/639 (1077 bp) on genomic DNA of *T. forsythia* ATCC 43037 Δ*Tanf_08365* mutant with integrated *ermF*. Primers 640/641 yield a 935-bp PCR fragment when using *T. forsythia* wild-type genomic DNA, whereas this fragment is absent on genomic DNA of the Δ*Tanf_08365* mutant confirming the loss of the gene; O´Gene Ruler 1 kb Plus DNA Ladder (Thermo Fisher Scientific) was used as a gene ladder.
